# From survival to lifelong care: The role of patient organisations in aortic dissection

**DOI:** 10.1016/j.ahjo.2026.100834

**Published:** 2026-07-08

**Authors:** Nimrat Grewal

**Affiliations:** a Department of Cardiothoracic Surgery, Amsterdam University Medical Center location AMC, Amsterdam, the Netherlands; b Aortic Institute at Yale-New Haven Hospital, Yale University School of Medicine, New Haven, United States

**Keywords:** Aortic dissection, Patient organisations, Continuity of care, Patient education, Psychosocial support

## Abstract

Survival after an acute aortic dissection (AAD) has improved significantly in recent decades. However, for most patients hospital discharge marks the beginning of lifelong surveillance rather than recovery. AAD survivors face persistent risks of late complications, strict blood pressure control, repeated imaging, and considerable psychological burden. Patient organisations may address these gaps by providing peer support, practical education, and continuity across healthcare settings. By translating medical recommendations into daily practice and facilitating engagement with care and research, such organisations can complement routine clinical follow-up. This need may be particularly relevant in healthcare systems with large and diverse populations, where continuity of specialist care is variable. Recognising patient organisations as partners in survivorship may contribute to more comprehensive, patient-centred management after aortic dissection.

## 1 Introduction

An acute aortic dissection (AAD) is defined as a tear in the largest vessel. AADs remain one of the most catestrophic cardiovascular emergencies with a mortality of more than 90% in the first year if left untreated [1]. Survival depends on rapid recognition and immediate treatment. Over the past decades, outcomes in the acute phase have improved due to better imaging, surgical techniques, and perioperative care [2]. As a result, more patients now survive the initial event.

What follows after survival, however, is less clearly defined. Many patients leave the hospital with the understanding that they have narrowly escaped death. However, there is limited insight into what living with a dissected aorta actually entails. Lifelong blood pressure control, repeated imaging, and the constant awareness of recurrence or complications become part of daily life [3]. These aspects of care are often only partly addressed during routine follow-up. This commentary discusses the role of patient organisations as complementary partners in long-term survivorship after aortic dissection, with a particular focus on continuity of care, education, and psychosocial support. This paper reflects on how patient organisations may complement specialised aortic care in long-term survivorship.

## 2 Aortic dissection does not end at discharge

For most patients, aortic dissection is sudden and unexpected. The acute episode is frequently remembered in vivid detail and is often described as traumatic. Several studies have shown that this experience has lasting psychological effects, including anxiety and persistent fear of recurrence [4], [5].

From a medical perspective, survival does not equate to resolution. The residual aorta remains at risk [6], and patients require lifelong surveillance. Even in those with stable imaging findings, uncertainty about the future is common. Long-term follow-up studies confirm that quality of life after dissection is frequently reduced, particularly due to limitations in physical confidence and ongoing mental stress [4], [5], [7].

Dissection also affects patients at an age when work, family responsibilities, and long-term planning matter. Questions about exercise, pregnancy, employment, and family screening are raised early but are not always revisited systematically during follow-up.

## 3 Gaps in post-dissection care

After discharge, care often becomes fragmented. Patients may transition from a tertiary centre to regional hospitals or primary care, where specific expertise in aortic disease is limited. Adherence to recommended imaging schedules and blood pressure targets is therefore variable, as shown in several observational studies [8]. At the same time, dedicated aortic surveillance programs have demonstrated substantially improved adherence to imaging follow-up and have been associated with improved long-term survival, highlighting the importance of structured survivorship pathways [9].

Patients frequently report that they are unsure which symptoms should prompt urgent medical attention and which can be observed. Many describe feeling responsible for monitoring their own condition without feeling adequately equipped to do so. These uncertainties contribute to anxiety and repeated emergency visits, sometimes for benign complaints, sometimes for delayed recognition of serious complications.

Questions regarding exercise and physical activity are particularly common after dissection, as patients frequently experience uncertainty regarding safe activity levels despite existing expert recommendations [10].

Psychological support is rarely structured. Anxiety, depressive symptoms, and post-traumatic stress–related complaints are common but often remain unspoken during cardiology or surgical follow-up visits [11]. Family members, who may have witnessed the acute event, are seldom included in follow-up conversations despite their central role in long-term support.

## 4 What patient organisations add

Patient organisations may complement, but not replace, specialised aortic follow-up programs by reinforcing education, continuity, and psychosocial support outside the hospital setting.

Their value lies less in replacing medical care and more in reinforcing it. Examples of existing initiatives include international awareness campaigns such as Think Aorta, as well as organisations including the John Ritter Foundation, Aortic Hope, and Aortic Dissection Awareness, which provide educational resources, advocacy, and peer support for patients affected by aortic disease.

Contact with and peer support from other patients who have experienced a dissection could help normalise fears and reduces isolation. Hearing how others manage blood pressure, imaging follow-up, and daily life provides reassurance that cannot easily be achieved in a clinical setting. For some patients, this peer contact lowers the threshold to seek professional help when psychological symptoms persist.

Education is another key contribution. Patient organisations often translate guideline-based recommendations into language that reflects everyday concerns [12]. Understanding and adherence among the patients can signifcantly be improved by practical explanations of why imaging is repeated, what blood pressure targets mean in daily life, and how to communicate a history of dissection to other healthcare providers.

In addition, patient organisations can help patients navigate the healthcare system. Simple tools such as written summaries of the diagnosis, previous interventions, and follow-up requirements support continuity of care, particularly in emergency settings or when patients change providers.

Finally, patient organisations increasingly play a role in research [4], [5]. By facilitating patient engagement and highlighting outcomes that matter to patients, such as quality of life and functional recovery, they contribute to a more complete understanding of long-term outcomes after dissection. Collaborative initiatives such as the Aortic Dissection Collaborative further illustrate how patient advocates can contribute to defining research priorities and patient-centred outcome measures in aortic disease [13].

## 5 Variability in survivorship care

Access to long-term survivorship care after aortic dissection varies considerably between healthcare systems and regions. Even in settings with advanced acute aortic care, continuity of specialist follow-up, access to imaging, rehabilitation, and psychosocial support may remain inconsistent. Survivorship challenges following dissection are therefore not limited to a single healthcare system, but represent broader issues in long-term cardiovascular care.

Patient organisations tailored to aortic dissection could complement existing care without requiring substantial resources. Experience from other settings suggests that even small-scale initiatives can support education, continuity, and psychosocial wellbeing when closely aligned with clinical practice.

## 6 Practical scope of support

A dissection-focused patient organisation can contribute by reinforcing and delivering information remotely, which already provided in the clinic. Clear written explanations of blood pressure targets, follow-up imaging intervals, and warning symptoms help patients retain and apply medical advice. Emergency summaries that document the diagnosis and prior treatment can be particularly useful when patients present to non-specialist services.

Peer support, when appropriately moderated, allows patients and families to share experiences while minimising the risk of misinformation. Providing information for family members, including when screening may be appropriate, addresses concerns that are frequently raised but not always fully explored during follow-up visits.

Structured rehabilitation programs may further support recovery after dissection by reinforcing cardiovascular risk reduction, supervised physical activity, and patient education. However, access to such programs remains variable across healthcare systems.

Collaboration with clinicians is essential to ensure medical accuracy and to align patient-led resources with standard care pathways.

## 7 Conclusion

**Fig. 1:**
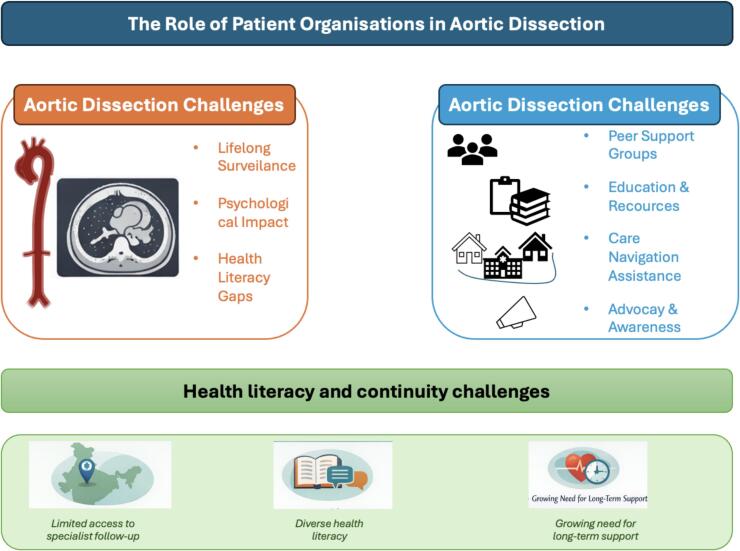
Conceptual overview of the role of patient organisations in long-term care after aortic dissection. Survivors face persistent challenges such as lifelong surveillance, psychological burden, and health literacy gaps. Patient organisations may complement specialised follow-up through peer support, education and resources, care navigation, and advocacy and awareness. These contributions are particularly relevant given cross-cutting challenges in health literacy and continuity of care, including limited access to specialist follow-up and a growing need for long-term support.

Surviving an aortic dissection marks the beginning of a chronic condition rather than the end of an acute event. Medical follow-up alone does not address all the challenges patients face in the years that follow. Patient organisations offer support in areas where routine care is limited, particularly education, continuity, and psychosocial wellbeing. Recognising their role as part of long-term dissection care reflects the realities of living with this condition Fig. 1.

## CRediT authorship contribution statement

**Nimrat Grewal:** Writing – original draft, Validation, Conceptualization.

## Declaration of competing interest

The authors declare that they have no known competing financial interests or personal relationships that could have appeared to influence the work reported in this paper.
